# Video Education Intervention in the Emergency Department

**DOI:** 10.5811/westjem.2022.9.57986

**Published:** 2022-12-09

**Authors:** Nancy Jacobson, Keli D. Coleman, Steven J. Weisman, Amy L. Drendel

**Affiliations:** * Medical College of Wisconsin, Department of Emergency Medicine, Milwaukee, Wisconsin; † Medical College of Wisconsin, Department of Pediatrics, Section of Emergency Medicine, Milwaukee, Wisconsin; ‡ Medical College of Wisconsin, Department of Anesthesiology, Milwaukee, Wisconsin

## Abstract

**Introduction:**

After discharge from the emergency department (ED), pain management challenges parents, who have been shown to undertreat their children’s pain. Our goal was to evaluate the effectiveness of a five-minute instructional video for parents on pain treatment in the home setting to address common misconceptions about home pediatric pain management.

**Methods:**

We conducted a randomized, single-blinded clinical trial of parents of children ages 1–18 years who presented with a painful condition, were evaluated, and were discharged home from a large, tertiary care pediatric ED. Parents were randomized to a pain management intervention video or an injury prevention control video. The primary outcome was the proportion of parents that gave their child pain medication at home after discharge. These data were recorded in a home pain diary and analyzed using the chi square test to determine significant difference. Parents’ knowledge about components of at-home pain treatment were tested before, immediately following, and two days after intervention. We used McNemar’s test statistic to compare incorrect pretest/correct post-test answers between intervention and control groups.

**Results:**

A total of 100 parents were enrolled: 59 parents watched the pain education video, and 41 the control video. Overall, 75% of parents completed follow-up, providing information about home medication use. Significantly more parents provided pain medication to their children after watching the educational video: 96% vs 80% (difference 16%; 95% CI 7.8–31.3%). Significantly more parents had correct pain treatment knowledge immediately following the educational video about pain scores (P = 0.04); the positive effects of analgesics (P <0.01); and pain medication misconceptions (P = 0.02). Most differences in knowledge remained two days after the video intervention.

**Conclusion:**

The five-minute educational video about home pain treatment viewed by parents in the ED prior to discharge significantly increased the proportion of children receiving pain medication at home as well as parents’ knowledge about at-home pain management.

## INTRODUCTION

Approximately 57% of children have pain on arrival to the emergency department (ED).^1^ Most children are discharged home with moderate or severe pain and require pain treatment.^2^–^5^ Injuries (eg, fractures, sprains, strains, contusions) are the most common cause of ED visits by children in pain. Fractures account for 10–25% of the injuries in children, and fracture pain is most severe 48 hours after discharge from the ED, making early at-home pain treatment particularly important.^6^,^7^ In a cross-sectional study, nearly 32% of parents reported dissatisfaction with the at-home pain management of their children with fracture pain when asked to recall their experiences during the prior week.^7^ The impact of parental pain management knowledge on at-home pain experience and satisfaction is not known.^8^,^9^

Parents’ knowledge of pain treatment is a priority since prior studies have shown parents lack pain management knowledge and underestimate and undertreat their children’s pain.^10^–^13^ Some parents report the belief that pain prevents further injuries and that minimizing medication use is optimal, analgesics are addictive, and analgesics work better the less they are used.^13^,^14^ Improving parents’ knowledge by providing structured guidance may optimize children’s home pain experience and is, therefore, essential to emergency medical care to promote the best patient outcomes.

Studies suggest that video instruction may be preferred by parents.^15^,^16^ Video guidance circumvents some ED challenges such as limited time, insufficient personnel, and literacy level of caregivers.^17^ Brief video education standardizes knowledge and avoids inconsistencies in information given by individual clinicians; using visual and auditory learning tools improves caregivers’ knowledge and enhances self-efficacy at hospital discharge.^18^–^20^ Cell phones, tablets, and computers are ubiquitous in the ED setting; so accessing an educational video can be quite simple. Structured information given both verbally and written with visual clues has improved recall and at-home compliance after discharge.^20^ However, there are no studies of the use of a video to enhance caregivers’ understanding of treatment of injuries after discharge.

Since many children with painful complaints are discharged home from the ED and are cared for by their parents, and parents are known to underestimate and undertreat pain, efforts should be made to reduce the pain experience at home. This is important because unrelieved severe pain can lead to an altered pain response, slower healing, anxiety, fear of medical encounters, decreased quality of life, and chronic pain.^21^–^23^ Increasing parents’ knowledge about pain management for their children may result in increased use of over-the-counter analgesics at home, which may translate into an improved pain experience for children. In this study we investigated whether an instructional pain treatment video shown to parents at ED discharge would increase their use of pain medication for their child at home and increase their knowledge about pain. We hypothesized more children would receive pain medication during the three days after discharge from the ED if their parents viewed the educational video in the ED. We also hypothesized that parental knowledge about pediatric pain management would improve after viewing an educational video in the ED.

## METHODS

### Study Design and Setting

This was a single-blind randomized clinical trial evaluating an instructional video intervention to improve parents’ pain knowledge and medication use in the home. This study was conducted between June–August 2011 in a children’s hospital Level I trauma center ED with an average annual census of 65,000 patients, with an average of ~180 pediatric visits each day. The study was reviewed and approved by the institutional review board. All parents and children provided informed consent/assent before enrollment. This clinical trial was registered with the National Institutes of Health (clinicaltrials.gov identifier NCT00520442).

**Population Health Research Capsule**

What do we already know about this issue?

*Most children with pain in the emergency department (ED) have ongoing pain that needs treatment after discharge. Studies show parents underestimate and undertreat children’s pain*.

What was the research question?

*Will an instructional video increase the proportion of children receiving pain medicine at home and improve parent knowledge?*

What was the major finding of the study?

*More parents provided medicine: 96% vs 80% and increased their knowledge for two days after the video presentation*.

How does this improve population health?

*An instructional video about pain management during the ED visit can change parents’ behavior and increase their knowledge about their child’s healthcare needs at home*.

### Population

A convenience sample of eligible parents of children ages 1–18 years presenting to the ED for painful chief complaints (including complaints of pain, injury, laceration, fracture, sprain, contusion, crush injuries, head injury, motor vehicle collision, burn, or non-traumatic painful injury), were approached for enrollment between 11 am and midnight, seven days a week. Parents were not eligible if they were not the legal guardian, or the child did not report pain at time of enrollment or had a chronic painful condition (eg, sickle cell disease, rheumatoid arthritis). Parents were ineligible if they were not English-speaking or were inaccessible by telephone for follow-up.

### Study Protocol

The figure summarizes the study protocol. After eligibility criteria were confirmed and consent was obtained, parents provided demographic information and completed a pain knowledge test to establish their baseline knowledge about pain treatment. Parents were randomized to the intervention educational video or control video but blinded to the study hypothesis. After evaluation and treatment in the ED, but prior to discharge, parents viewed the assigned video. Immediately after watching the video, they completed the same pain knowledge test to evaluate immediate change in knowledge. All parents were then instructed on the use of an at-home pain diary to record their child’s reported pain and pain medication use. On the second day after discharge, parents completed the same pain knowledge test to evaluate knowledge retention. The data from the at-home pain diary and answers to the knowledge test were all collected via daily standard follow-up telephone calls made during the first 48 hours after the ED visit. This timeline was chosen since the worst pain is typically experienced within 48 hours after ED discharge.^7^

All diagnosis and treatment decisions in the ED were made by board-certified pediatric emergency medicine specialists or fellows. Children received the usual care for their painful conditions in the ED. The treating physician and nurse provided the usual discharge instructions based on the discharge diagnosis to all families at time of discharge regardless of group assignment and were blinded to enrollment group. The ED did not have a standardized discharge recommendation for children with pain.

### Randomization and Intervention

A random number table was used to assign parents to either an intervention or control video. The treating physician, nurse, patient, and parent were blinded to the video assignment and study hypothesis. The intervention video was a five-minute educational video designed to address outpatient pediatric pain and its management. The video was narrated by a pediatric emergency physician (AD) and was developed to incorporate known deficits in parent knowledge.^24^ The control video was a five-minute video about pediatric fall and injury prevention.^25^

### Methods of Measurement

#### Parent characteristics

Parent’s age, race, gender, and level of education were collected using a parent report on a standard survey at time of enrollment.

#### Child characteristics

Child’s age, pain score at arrival and discharge, and painful conditions were abstracted from the patient record. The child’s pain in the ED was measured with the pain tool appropriate for age with a score range of 0–10.

#### Analgesic Use

The primary outcome of interest was parent’s use of any analgesic for their child at home during the first 48 hours after discharge from the ED, including ibuprofen or acetaminophen or prescribed analgesics. This outcome was chosen as any increase would represent a change in the parent’s home management of pain. Parents used an at-home pain diary, previously developed for prior follow-up studies in our ED, that detailed time and type of home analgesic medication to facilitate verbal report at the daily telephone follow-up call.^5^

#### Pain Knowledge Assessment

Parents completed a pain knowledge test, a seven-question true-or-false quiz that focused on common knowledge gaps in pediatric pain management including pain awareness, pain assessment, and pain medication effects (Table 1). Three questions queried knowledge about fall and injury prevention, which was the focus of the control video. Items in the pain knowledge test were derived from a prior qualitative study of pain investigating parents’ knowledge, behaviors, and attitudes regarding at-home pain treatment.^23^ Parents completed this test before viewing the video (N = 100), after viewing the video but prior to discharge (N = 100), and 48 hours after discharge from the ED (N =75; 50 were in the intervention group). Test results were never discussed with the family.

### Data Analysis

This was a superiority study, designed to detect a 35% relative improvement in the primary outcome: the parent’s administration of pain medication to their child at home during the first 48 hours. An increase in the use of at-home pain medication by parents was defined as the parent’s use of at least one dose of any pain medication. Based on prior research, use of any at-home pain medication by parents was estimated to be 60%, and a 35% improvement in this rate was felt to be clinically important.^26^ Improvement by 35% would require 25 patients in each group to obtain an alpha = 0.05 and a beta = 0.20. A priori subgroup exploration was planned to investigate differential medication use when comparing younger to older children (cut-points of 6 years and 12 years), gender, race/ethnicity, and higher ED pain scores (score of 4 or greater).

We used descriptive statistics to analyze the demographic data. The intention-to-treat model was used to analyze outcomes. We compared increased pain medication use using chi-square test. The knowledge assessment results pre-video were combined for the intervention and control group. The knowledge assessment results for each of the three assessment periods were tabulated for the intervention and control group separately. We assessed the effect of the video intervention on parent knowledge using the McNemar test to compare incorrect pretest/correct post-test answers between intervention and control groups. The McNemar test was also used to examine the effect of the video on retained knowledge by comparing parents’ pain knowledge before the video and 48 hours after discharge. Each question was analyzed independently. We explored the effect of the video intervention on the child’s pain experience using the *t*-test to evaluate the median difference in pain scores, comparing the two groups.

## RESULTS

### Characteristics of the Subjects

During study enrollment, 157 eligible parents and their children were approached for participation in the study; 57 refused, leaving 100 parents and their children enrolled and randomized (Figure). We had excellent follow-up, with 75% of participants completing the 48-hour phone follow-up for knowledge retention and pain score reporting. For the intervention and control group, parents who were lost to follow-up were not different with respect to demographic characteristics (parent gender, age, race/ethnicity, education, or child’s age, pain on arrival and discharge), baseline pain knowledge, or knowledge after viewing the video. Baseline characteristics for the two groups are shown in Table 2.

Mostly mothers were enrolled, and the distribution of race/ethnicity and level of education is comparable to what is seen in this ED. Pain intensity experienced by children in the intervention and control groups was similar on arrival and after discharge from the ED. Painful injury complaints of children enrolled in the study included the following: fractures; sprains; lacerations; burns; motor vehicle collisions; contusions; and non-traumatic painful injuries.

### Analgesic Use

The primary outcome was the proportion of parents that gave their child any medication in the first 48 hours after discharge from the ED. Significantly more parents provided pain medication if they viewed the intervention video (96%) compared to parents who viewed the control video (80%) (difference: 16%; 95% CI 7.8–31.3%). Planned subgroup analyses were performed to identify children less likely to receive any analgesic at home. No difference was found in the proportion of children given any medication when comparing younger to older children, gender, race/ethnicity, or arrival and discharge pain scores of 4 or greater (moderate/severe pain).

### Parent Knowledge Assessment

See Table 3. Baseline knowledge was similar in the two groups. For both the intervention and control group together prior to viewing the video, many parents (85%) were aware that children do experience pain after they go home from the ED (question #1) and pain scores can measure pain for kids (86%) (question #2). Parents’ knowledge about the effects of pain medication was more variable. Nearly all parents (97%) knew that children who use pain medication will not become addicted (question #3), and many (87%) knew that using pain medication is not the only way to effectively treat pain (question #7). However, a knowledge gap was noted for the positive effects of pain medications: only 60% of parents knew using pain medication can get children back to normal activities more quickly (question #5 and only 63% knew medications can help children heal better (question #6). Few parents (31%) understood that pain medication does not hide underlying problems (question #4).

The proportion of parents with correct answers to the knowledge questions immediately after the intervention video was viewed are shown for both groups in Table 3. Differences in the proportion of parents that initially provided the wrong answer and then had the correct answer after the video when comparing the two groups are shown as a *P*-value. Significant improvements were found in parents’ knowledge about using a pain score for children (question #2), the positive effects of pain medication (questions #5 and #6), and the understanding that pain medications don’t hide underlying problems (question #4). However, awareness for pain experience after the ED (question #1), knowledge that children would not become addicted to pain medication (question #3), and an understanding that there are alternative ways of treating pain (question #7) remained high but were not significantly different.

Parents’ retention of the knowledge improvements two days after watching the video is also shown for both groups in Table 3. Differences in the number of parents that initially provided the wrong answer and then had the correct answer after the video when comparing the two groups are shown. The proportion of parents who knew that children experience pain after they go home from the ED significantly improved in the intervention group (question #1). Although this improvement was not apparent immediately after the video was viewed in the ED, it was noted at 48-hour follow-up. The significant improvements in parents’ knowledge about using pain scores (question #2) and the positive effects of the pain medications (questions #5 and #6) remained at 48 hours after ED discharge. The significant improvement in the understanding that pain medication will not hide underlying problems (question #4) that was apparent immediately after the video was viewed was no longer found.

## DISCUSSION

At-home pain treatment by parents for children has been shown to be inadequate.^3^–^5^,^12^,^13^,^27^–^31^ This study provides evidence to support the use of an instructional five-minute pain treatment video for parents in the ED setting to increase at-home pain treatment and parent knowledge. After viewing the video, a significantly higher proportion of parents administered pain medications for their children’s pain, and significant improvements in parents’ knowledge, particularly for the use of pain assessments and the positive aspects of using pain medications for children were shown. Current literature shows at-home pain treatment by parents for children is inadequate in many cases. This simple video presentation that can be implemented in the ED setting may help to improve care for children. This is a first step in the development of an ED intervention to optimize at-home pain treatment for children.

Videos,^10^,^15^,^18^,^32^–^34^ online videos,^9^,^35^ and web-based modules^9^,^35^ improved parents’ knowledge of pediatric disease management and the comprehension of discharge instructions by caregivers of children presenting to the ED for pediatric gastroenteritis^32^,^33^ bronchiolitis,^33^ and fever. 33,34 However, very few studies have been conducted using digital media as an intervention in pediatric fractures and painful injuries as a method to improve caregivers’ knowledge.

Digital media, which included web-based modules,^9^ videos,^9^,^10^,^18^ and mobile discharge instructions videos,^36^ improved the understanding of pain management and discharge instructions for fractures and painful injuries. Bloch and Bloch^18^ enrolled parents of children (29 days–18 years) in the ED with a number of chief complaints to determine whether video discharge instructions as an adjunct to standard written questions would improve caregivers’ comprehension of the children’s ED visits, medical plans, and follow-up instructions. The video discharge instructions significantly improved caregivers’ understanding in the ED and 2–5 days after discharge for children with non-painful complaints. Uniquely, our study enrolled children with painful chief complaints to evaluate the impact of the video not only to increase caregivers’ understanding of how to treat painful injuries in the home setting, but perhaps, more importantly, to affect the pain medicine administered to their children in the at-home setting. Our study showed a significant improvement in caregivers’ pain management knowledge, which is consistent with work reported by other investigators.^9^,^10^,^35^

Prior studies have shown that children with a fracture often do not receive treatment for pain.^37^–^40^ For example, of children discharged home from the ED with a bone fracture, 30% received no more than one dose of pain medication each day after the injury, despite reported high pain scores.^11^ In a clinical trial comparing analgesic effectiveness in the at-home setting, only 48% of children with moderate or severe fracture pain always received pain medication from their parents.^41^ This study showed the video intervention impacted at-home care for children. No other studies have shown that an ED intervention changes at-home actions by parents. This is a first step in the development of an intervention that may optimize at-home pain treatment.

There is no widely accepted discharge instruction standard for children, which may hinder efforts to improve parents’ knowledge after ED discharge.^42^ Discharge instructions are an essential factor in the ED visit aftercare.^32^ Some barriers to optimizing this process may be time constraints and variable discharge instructions^43^,^44^ and poor-quality instructions.^45^,^46^ Also, caregivers’ comprehension, recall of treatment plan, and follow-up plan are known to be inadequate.^46^,^47^ Literacy may also play a role in the lack of comprehension of the discharge instructions.^17^ Incomplete understanding of the discharge instructions may lead to incorrect treatment after discharge, readmissions, and a higher rate of dissatisfaction with care.^32^ Understanding of and compliance with discharge instructions has become more important as the management of more acute conditions is being shifted to the outpatient setting.^47^ In this regard, the pain management video standardized the information parents received. The increase in parents’ knowledge of pain management during painful injuries suggests that the video increased knowledge, which may be a key driver in optimizing at-home pain treatment.

Parents play a critical role in the treatment of a child’s pain in the at-home setting. Their decisions and behaviors are key factors in the child’s pain experience that are likely guided by their knowledge of pain management. It is not known why parents are not using pain medication as advised at home. It has been hypothesized that parents underestimate their child’s susceptibility to and severity of pain in the at-home setting, but the causality has never been prospectively investigated.^11^–^13^ The perceived risks and benefits of analgesics have also been hypothesized to affect parents’ decisions, as demonstrated by a number of myths reported by parents, including the belief that an analgesic might mask symptoms or that medications should only be used as a last resort.^41^,^48^,^49^ Adverse effects and addiction potential are also concerns to parents.

Given the complexity of the pain experience, several factors likely account for inadequate at-home pain treatment for children. A parent’s decision to administer analgesics is the central behavioral determinant of the child’s pain experience, and this decision is shaped by the parent’s knowledge and experiences. This is one of the few investigations of an ED educational video to impact this pivotal decision-making process. This distinctive, evidence-based intervention was successful in significantly improving parents’ use of analgesics for pain for their children at home and has the potential to be a first step toward improving the at-home pain experience for children.

## LIMITATIONS

Not all eligible patients were recruited due to the convenience sampling. This study was single blinded because a single researcher (NJ) provided the video and collected the outcomes. Another limitation is that our study could not discern whether the increase in the use of and knowledge about pain medication was due to the daily phone calls to collect outcomes (Hawthorne effect). Further, the study was conducted at a single teaching hospital, and non-English speakers and lack of phone for follow-up were excluded, possibly limiting its generalizability. Outcomes were collected within 48 hours after ED discharge when pain is generally most severe,^6^,^7^ so it is not known whether this intervention impacted pain experienced after this timeframe. Finally, it is not known whether these increases in pain medication administered impacted the child’s pain experience.

## CONCLUSION

A five-minute instructional pain-treatment video shown to parents during the ED visit increased parents’ use of pain medication for their child at home. Further, the instructional video increased parents’ knowledge of pediatric pain management.

## Figures and Tables

**Figure:**
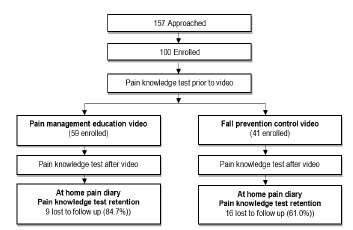
Summary of the study protocol.

**Table 1:** Pain knowledge test.

Brain injury is the #1 killer of children in the US today. (TRUE)* Most children do NOT experience pain after they go home from the emergency department. (FALSE) Pain scores can help measure pain for kids and pain scores of 4 or more should be treated. (TRUE) Children who use pain medications will become addicted. (FALSE) Most infant injuries occur when a parent leaves the child alone in a room. (FALSE) * Pain medications can hide underlying problems. (FALSE) Using pain medications after painful injuries can get children back to normal activities quicker. (TRUE) Using pain medications can help children heal better. (TRUE) Window screens are generally secure enough to hold a child inside a house. (FALSE) * Using pain medication is the only way to effectively treat pain. (FALSE)

* Questions identified with an asterisk were included to test knowledge gained about falls prevention that was the focus of the control video that was viewed.

*US*, United States

**Table 2:** Baseline characteristics for the two groups.

Characteristics	Intervention n (%) N = 59	Control n (%) N = 41
Parent Gender – female	50 (85%)	35 (85%)
Parent Age		
Mean (years)	36.2	36.2
Range (years)	20–62	20–62
Parent Race /Ethnicity		
White	31 (52%)	24 (58%)
Asian	0 (0%)	3 (7%)
Black	16 (27%)	6 (15%)
Hispanic	9 (15%)	5 (12%)
Other	3 (5%)	3 (7%)
Parent Education		
Some high school	7 (12%)	4 (10%)
High school graduate	12 (21%)	8 (20%)
Some college	11 (19%)	11 (27%)
College graduate	17 (29%)	11 (27)
Graduate studies	11 (19%)	7 (17%)
Child’s Age		
Mean (years)	7.5	7.5
Range (years)	1–18	1–18
Child’s Pain Experience: (median score)		
ED arrival	5	4
ED discharge	2	2

*ED*, emergency department.

**Table 3:** Parent knowledge assessment.

True/ false Statement	Pre-video		Post-video			2-day retention		
		
	Intervention	Control	Intervention	Control	p-value	Intervention	Control	p-value
Awareness								
1. Most children do NOT experience pain after they go home from the ED. (false)⁰	84.7%	85.4%	81.4%	75.6%	0.49	95.9%	79.1%	0.02*
Assessment								
2. Pain scores can help measure pain for kids and pain scores of 4 or more should be treated. (true)Φ	88.1%	82.9%	100.0%	83.0%	0.001*	100.0%	91.7%	0.04*
Pain								
3. Children that use pain medications will become addicted. (false)	96.6%	97.6%	98.3%	95.1%	0.36	93.9%	95.8%	0.73
4. Pain medications can hide underlying problems. (false)⋄	37.9%	24.4%	61.0%	34.2%	0.02*	57.1%	37.5%	0.12
5. Using pain medications after painful injuries can get children back to normal activities quicker. (true)Φ	57.6%	63.4%	98.3%	68.3%	<0.00*	93.9%	66.7%	0.002*
6. Using pain medications can help children heal better. (true)Φ	64.4%	61.0%	96.6%	56.1%	<0.00*	93.9%	58.3%	<0.00*
7. Using pain medication is the only way to effectively treat pain. (false)	87.9%	85.4%	79.7%	82.9%	0.69	92.8%	87.5%	0.55

This table reports the proportion of parents with correct answers. McNemar’s test and associated P-value were used to determine likelihood that a parent who changes from an incorrect answer to a correct answer on the pain knowledge test belongs to the intervention group.

Statements marked with an Φ were significantly more likely to be answered correctly in the intervention group during both assessments.

Statements with an ⋄ were significantly more likely to be answered correctly in the intervention group only immediately after the video was viewed.

Statements marked with an ⁰were significantly more likely to be answered correctly in the intervention group only 2 days after the video was viewed.

* denotes statistically significant changes in correct answers compared to Pre-video.
